# Supplementary material for: The effect of intrapartum prolonged oxygen exposure on fetal metabolic status: secondary analysis from a randomized controlled trial
Source: Front Endocrinol (Lausanne). 2023 Jun 27;14:1204956. doi: 10.3389/fendo.2023.1204956 (PMC10335765; doi:10.3389/fendo.2023.1204956)
Supplement: Supplementary file 3 [file DataSheet_3.zip › Result-X101SC21092977-Z01-J001-B1-42/1.MetQuant-QC/Samples_QC_neg-PCA.pdf]

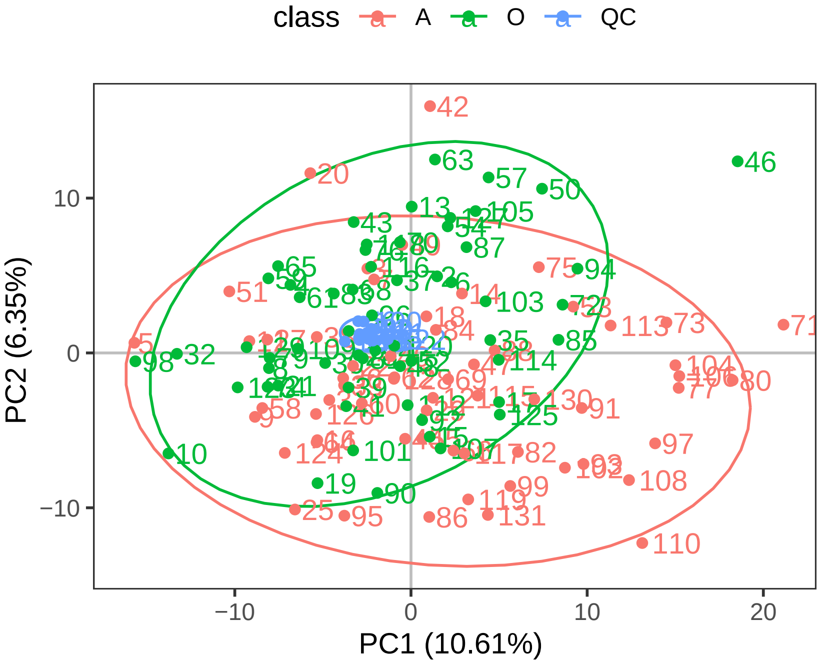

class
A
O
QC
PC1 (10.61%)
PC2 (6.35%)
−10
0
10
20
42
63
46
57
50
20
13
105
43
87
75
94
65
51
61
14
103
18
113
73
71
84
35
85
5
98
32
114
104
80
77
58
126
91
124
101
10
97
82
108
19
90
99
119
25
95
86
131
110
